# Mild Positive Pressure Improves the Efficacy of Benzalkonium Chloride against *Staphylococcus aureus* Biofilm

**DOI:** 10.3390/bioengineering9090461

**Published:** 2022-09-09

**Authors:** Shamaila Tahir, Sarah Emanuel, David W. Inglis, Karen Vickery, Anand K. Deva, Honghua Hu

**Affiliations:** 1 Surgical Infection Research Group, Faculty of Medicine, Health and Human Sciences, Macquarie University, Sydney, NSW 2109, Australia; 2 School of Engineering, Macquarie University, Sydney, NSW 2109, Australia

**Keywords:** disinfectant, positive pressure, benzalkonium chloride, synergy, *Staphylococcus aureus*, biofilm

## Abstract

Current protocols using liquid disinfectants to disinfect heat-sensitive hospital items frequently fail, as evidenced by the continued isolation of bacteria following decontamination. The contamination is, in part, due to biofilm formation. We hypothesize that mild positive pressure (PP) will disrupt this biofilm structure and improve liquid disinfectant/detergent penetration to biofilm bacteria for improved killing. *Staphylococcus aureus* biofilm, grown on polycarbonate coupons in the biofilm reactor under shear at 35 °C for 3 days, was treated for 10 min and 60 min with various dilutions of benzalkonium chloride without PP at 1 atmosphere (atm), and with PP at 3, 5, 7, and 10 atm. The effect on biofilm and residual bacterial viability was determined by standard plate counts, confocal laser scanning microscopy, and scanning electron microscopy. Combined use of benzalkonium chloride and PP up to 10 atm significantly increased biofilm killing up to 4.27 logs, as compared to the treatment using disinfectant alone. Microscopy results were consistent with the viability plate count results. PP improved disinfectant efficacy against bacterial biofilm. The use of mild PP is possible in many flow situations or if equipment/contaminated surfaces can be placed in a pressure chamber.

## 1. Introduction

Pathogens that can form biofilms frequently cause healthcare-associated infections (HAI) which impact patient morbidity and mortality. Transmission of pathogens and development of HAIs is a complex interplay between healthcare workers, patients, environmental contamination, medical devices/implants, antimicrobial regimens, and other infection control measures. The hospital environment, equipment, and reusable medical devices are known to be a source of HAI pathogens, including multidrug resistance organisms (MDROs) [1]. Adequate cleaning and disinfection are necessary to halt the cycle of cross-contamination. Unfortunately, current cleaning and disinfection are frequently suboptimal. Suboptimal decontamination leaves the organic matter in situ which often leads to biofilm formation [2,3].

In one study, 9% of reusable tourniquets were found contaminated with *Acinetobacter baumannii* and methicillin-resistant *Staphylococcus aureus* while another 32% of stethoscopes were contaminated with MDRO. These items may serve as potential sources of pathogen transmission [4,5].

Biofilm is a complex multilayered community of micro-colonies where bacterial phenotype adjusts to the limited availability of oxygen and nutrients [6]. Protected within the coating of exopolymeric substances (EPS), biofilm bacteria stay safe against adverse environmental conditions, displaying increased tolerance to desiccation, detergents, and disinfectants [7].

Removing and/or killing biofilm contaminating surfaces has been a significant area of interest for researchers due to its challenges. While detergents effectively remove planktonic bacteria, blood, and dirt from surfaces, they are relatively ineffective at removing biofilm and, in some cases, have no efficacy at all [8,9]. Similarly, disinfectants are very good at killing planktonic bacteria but have been shown to have decreased efficacy against biofilms [10]. The effectiveness of disinfectants is diminished even more if the biofilm is formed on channels within endoscopes that are subjected to multiple cycles of use and decontamination [11,12]. Similarly, biofilms formed on dry surfaces, where they are subjected to low water availability, are also exceedingly difficult to eradicate [13]. We demonstrated that some bacteria within *S. aureus* dry surface biofilms survived a 10 min treatment with sodium hypochlorite at concentrations of up to 20 times that used for hospital disinfection [14]. We demonstrated that survived cells lacked genetic change and so the dry surface biofilm tolerance to chlorine was due to the biofilm lifestyle [14], especially the very thick EPS of the *S. aureus* dry surface biofilm [15], creating a diffusion barrier around the bacterial cells. We suggested that physical wiping could improve disinfection action by dispersing the biofilm, thus decreasing the diffusion distance and improving the exposure of biofilm cells to the disinfectant.

The in vitro wound model combined with topical negative pressure therapy published by our research group [16,17] showed that negative pressure therapy compressed traditional *Pseudomonas aeruginosa* and *S. aureus* hydrated biofilm, reducing the biofilm thickness, thus reducing the diffusion distance for antiseptics to penetrate the biofilm, and resulted in improved bacterial cell killing in the biofilm. Ultrahigh pressures of 100 Mpa to 800 Mpa, equivalent to 986.9−7895 atmospheres (atm), have also been shown to inactivate bacteria when used to decontaminate food prepared for storage purposes [18]. In the current paper, we hypothesize that the penetration of commonly used fluid disinfectants can be improved with the synergistic use of mildly increased atmospheric pressures to disrupt biofilm structure for the enhanced killing of biofilm cells. Here, we tested the synergistic action of different mild positive pressure with various dilutions of a common disinfectant benzalkonium chloride against a *S. aureus* biofilm.

## 2. Materials and Methods

### 2.1. Staphylococcus aureus Biofilm Production

A *S. aureus* biofilm was used as a model organism to test the efficacy of using a disinfectant to kill biofilm cells in the presence or absence of PP. A CDC bioreactor (CBR, BioSurface Technologies Corp, Bozeman, MT, USA) was used to grow the inter-experimental reproducible *S. aureus* biofilm ATCC 25923 on 24 removable polycarbonate coupons.

The biofilm was grown over 3 days using batch phase (50% (15 g/L) Tryptone Soya Broth TSB) for 24 h, then the growth media in CBR was replaced with fresh 20% (6 g/L) TSB, followed by a flow-through phase (20% TSB introduced into the bioreactor at a flow rate of 80 mL/h) for 48 h under shear at 130 rpm at 35 °C. At the end of 3 days, the biofilm-covered coupons were gently washed in phosphate-buffered saline (PBS) three times to remove planktonic and loosely attached bacteria.

### 2.2. Strategy for Proof of Concept

The purpose of the study is to demonstrate the synergy effect of positive pressure and the disinfectant as proof of concept.

To show the synergy between the disinfectant action and the application of positive pressure, the tested disinfectant concentration had to be lower than that required to kill the biofilm without the addition of pressure. 

The test disinfectants were benzalkonium chloride 200 g/L [Bactex^®^ Concentrate quaternary ammonium compound (QAC), pH neutral hospital-grade disinfectant (Whiteley Cooperation, North Sydney, NSW, Australia) – normal in-use concentration is 2 g/L or a 1/100 dilution of Bactex^®^ concentrate].

#### Preliminary Titration of Disinfectants

Biofilm-coated coupons were treated with 2 mL of the prepared chemical dilutions (n = 6/dilution) for 10 min or 60 min of contact time. On completion of the test duration, coupons were washed three times with 3% bovine serum in PBS to remove and inactivate residual disinfectant. Coupons were placed in 4 mL of PBS and subjected to sonication at 43 kHz for 5 min, followed by 2 min of vigorous shaking to disrupt and release bacteria from the biofilm, followed by serial 10-fold dilutions and standard plate culture for colony forming units (CFU) determination.

Preliminary experiments with the recommended in-use concentration of benzalkonium chloride QAC (2 g/L) killed all the bacterial cells within the *S. aureus* biofilm. At this concentration, we were unable to demonstrate synergy between antiseptics and PP, therefore, we needed to test a concentration of antiseptic that failed to kill all biofilm cells.

The disinfectants were serially diluted in sterile water and the concentrations of benzalkonium chloride tested were 4 g/L, 2 g/L, 200 mg/L, 100 mg/L, 40 mg/L, 20 mg/L, and 10 mg/L, which were 200%, 100%, 10%, 5%, 2%, 1%, and 0.5% of the in-use concentration (IUC), respectively.

Untreated positive control coupons (n = 24 in total) had an average of Log_10_ 7.16 ± 0.39 CFU/coupon. Coupons treated with 200 mg/L (10% of the in-use concentration) resulted in a 4Log_10_ reduction in titer, but growth was easily detected (data not shown). Therefore, to test for synergism between disinfectants and PP, 200 mg/L was the maximum concentration tested.

### 2.3. Combined Positive Pressure and Disinfectant Efficacy Testing

Six biofilm-coated coupons were incubated in water and served as a positive control.

To test for synergism between benzalkonium chloride QAC and positive pressure (PP) treatments, disinfectant dilutions just lower than the one showing the complete killing of biofilm bacteria (sub-lethal doses) were selected. Coupons were placed in 2 mL of diluted disinfectant or water (positive control coupons, n = 6; positive pressure coupons) for 10- or 60-min treatments, as described below.

Based on the results obtained in the preliminary titration, the benzalkonium chloride QAC dilutions tested were 200 mg/L, 100 mg/L, 40 mg/L, and 20 mg/L, which were 10%, 5%, 2%, and 1% IUC, respectively.

**Figure 1:**
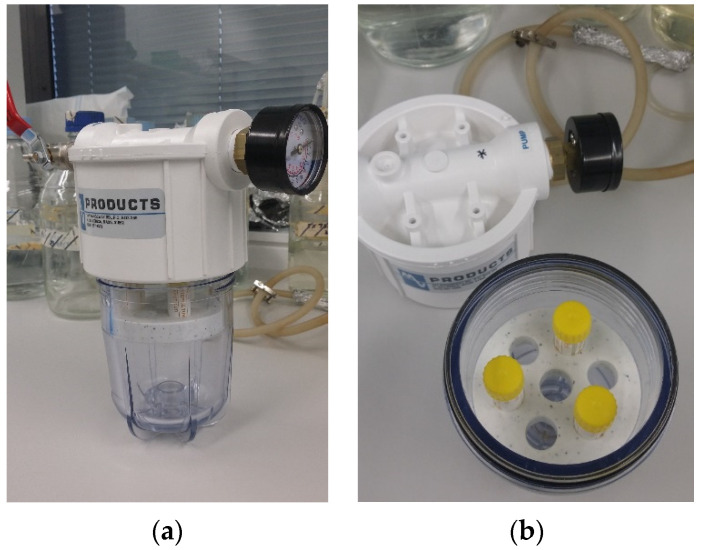
Positive pressure (PP) chamber. (**a**) From outside and (**b**) from inside with 5 mL sterile serum tubes with loose lids containing biofilm coated coupons for various treatments.

(1) Diluted test disinfectant only without PP (1 atm).
(2) PP only, at 3, 5, 7, and 10 atm in a positive pressure chamber designed and custom made by Dr. David Inglis (Figure 1).
(3) Combined disinfectant and PP at 3, 5, 7, and 10 atm.

The positive control coupons, PP, and/or benzalkonium chloride QAC treated coupons were individually placed in 2 mL of PBS and sonicated in an ultrasonic bath (Soniclean; Dudley Park, SA, Australia) for 10 min at 42–47 kHz, followed by a 2 min vortex. The viability of bacterial cells was counted by standard plate culture and colony forming units (CFU).

CFU log reduction was calculated as the CFU Log_10_ value of positive control minus the CFU Log_10_ value in each treatment condition. Each treatment condition was tested in triplicates in two independent experiments. The standard deviation was calculated from the CFU Log_10_ value of the six replicates in each treatment condition.

### 2.4. Confocal Laser Scanning Microscopy

Additional polycarbonate coupons were used for confocal laser scanning microscopy (CLSM) and scanning electron microscopy (SEM) for the positive control and each treatment condition, i.e., disinfectant only, PP only, and combined disinfectant and PP at 10 atm for 10 min. Following each treatment, the coupon was washed as above with PBS and then stained with a LIVE/DEAD BacLight bacterial viability kit (Molecular Probes, Invitrogen, Carlsbad, CA, USA), as per the manufacturer’s instructions. Live bacteria were stained green and dead bacteria were stained red. Biofilm grown on the coupon was then fixed for one hour with 4% paraformaldehyde and washed three times with PBS for 10 min each. Images were scanned for 2D and 3D imaging on a Fluoview FV1000 CLSM (Olympus Cooperation, Shinjuku, Japan) within 24 to 48 h of staining.

The 3D Images were built with 0.2 µm optical sections and analyzed for average thickness, biofilm mass, and percentage of viable cells using the IMARIS 7.7.2 software (Bitplane, Zurich, Switzerland) and the ImageJ program (Java application for scientific image processing, https://imagej.nih.gov/ij/ (accessed on 25 August 2022), U. S. National Institutes of Health, Bethesda, Maryland, USA), as described in our previous publication [17].

### 2.5. Scanning Electron Microscopy

Following CLSM imaging, the coupons were dehydrated through serial dilutions of ethanol and hexamethyldisilazane (HMDS, Sigma, St. Louis, MO, USA) for 10 min each, aspirated dry, and air-dried for more than 48 h. The coupons with a dehydrated biofilm were then mounted on specimen stubs, gold coated, and examined at low and high magnification using JOEL 6480LA scanning electron microscopy (JOEL, Tokyo, Japan).

### 2.6. Statistical Analysis

ANOVA (univariate analysis of variance), followed by Dunnett’s multiple comparisons test, was performed to check significant differences in the biofilm bacteria CFU log reduction, biofilm thickness, and biomass of various treatment groups in comparison to the control group using GraphPad Prism version 9.3.1 for Windows (GraphPad Software, San Diego, CA, USA, www.graphpad.com, accessed on 25 August 2022).

## 3. Results

Enhanced killing of bacterial cells occurred when in-vitro grown biofilm was exposed to PP along with chemical treatment, as compared to the treatment with test disinfectant or PP alone.

### 3.1. Effect of Positive Pressure on Benzalkonium Chloride Treatment against Staphylococcus aureus Biofilm

**Figure 2:**
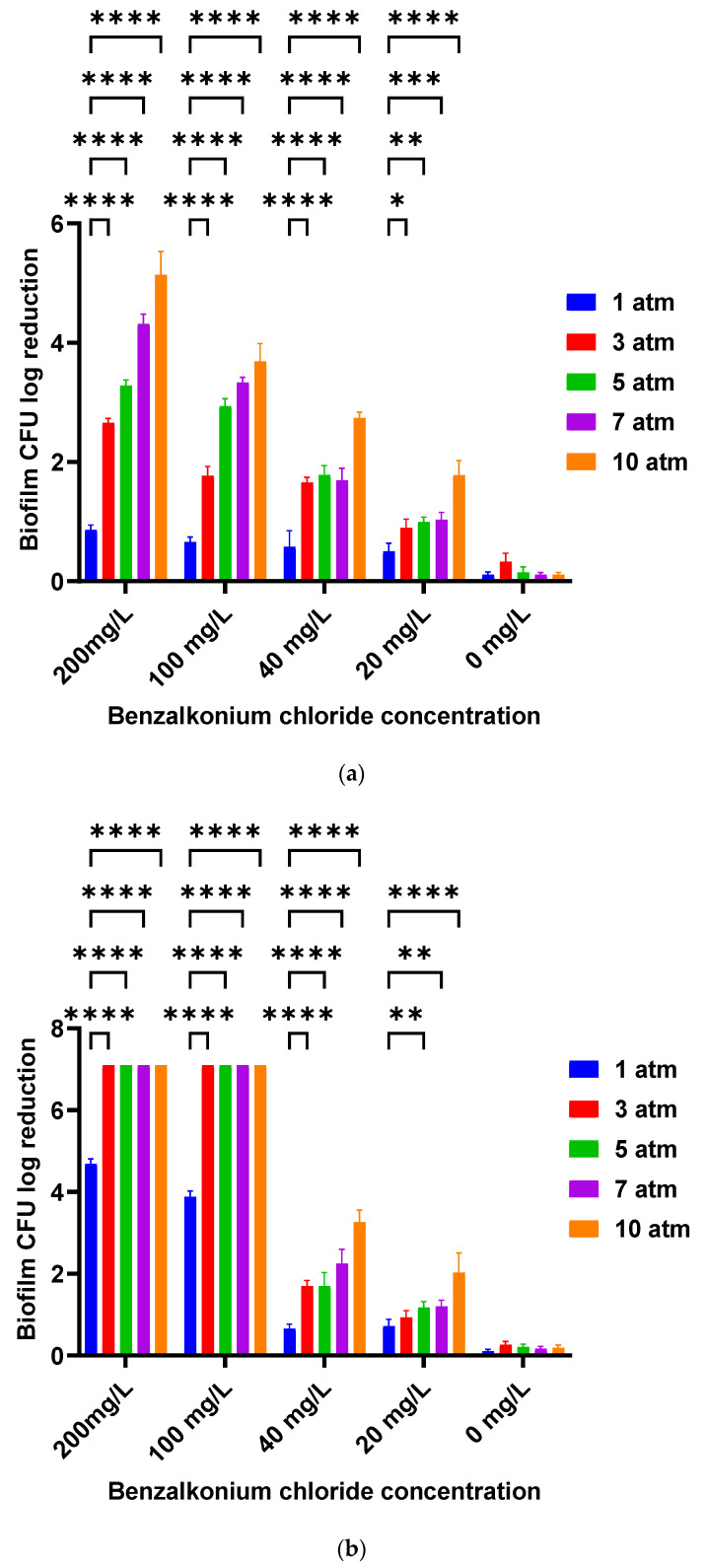
Biofilm CFU Log_10_ reduction after treatment with benzalkonium chloride in various concentrations without PP (at 1 atm) and with PP (at 3, 5, 7, and 10 atm) for 10 min (**a**) and 60 min (**b**). * *p* < 0.05, ** *p* < 0.01, *** *p* < 0.001, **** *p* < 0.0001.

Treatment with 10% IUC (200 mg/L) or 5% IUC (100 mg/L) of benzalkonium chloride only without PP (1 atm) for 10 min resulted in a 0.86 ± 0.07 or 0.67 ± 0.08 log_10_ reduction in biofilm CFU, respectively. Subjecting biofilm to 3, 5, 7, and 10 atm of PP for 10 min during the 200 mg/L (10% IUC) or 100 mg/L (5% IUC) benzalkonium chloride treatment resulted in a significant increase in the killing of the *S. aureus* biofilm (*p* < 0.001) (Figure 2a). Applying 10 atm of PP for a 10 min treatment of 10% IUC (200 mg/L) or 5% IUC (100 mg/L) benzalkonium chloride resulted in a 5.13 ± 0.39 or 3.69 ± 0.51 log_10_ reduction, respectively (*p* < 0.001) (Figure 2a).

Treatment with 10% IUC (200 mg/L) or 5% IUC (100 mg/L) of benzalkonium chloride only without PP for 60 min resulted in a 4.69 ± 0.12 or 3.88 ± 0.14 log_10_ reduction in biofilm CFU, respectively. Subjecting the biofilm to 3, 5, 7, and 10 atm of PP during the 200 mg/L (10% IUC) or 100 mg/L (5% IUC) benzalkonium chloride 60 min treatment resulted in a significant increase in the killing of the *S. aureus* biofilm (*p* < 0.001) (Figure 2b). Applying 10 atm of PP during the 60 min treatment of 10% IUC (200 mg/L) and 5% IUC (100 mg/L) benzalkonium chloride resulted in the complete killing of biofilm cells (*p* < 0.001) (Figure 2b).

Even only 1% IUC (20 mg/L) and 2% IUC (40 mg/L) of benzalkonium chloride resulted in significantly greater log reductions when combined with PP of 10 atm. However, increasing the contact time from 10 min to 60 min did not show increased biofilm killing (Figure 2a,b).

There was no significant reduction in CFU when *S. aureus* biofilm coated coupons were treated only with PP of 3, 5, 7, and 10 atm for 10 or 60 min (*p* > 0.05) without antiseptics or disinfectants (Figure 2a,b). This shows that mild PP itself does not kill the biofilm bacterial cells, but it facilitates the process of benzalkonium chloride killing the bacterial cells inside the biofilms.

### 3.2. Confocal Laser Scanning Microscopy and Scanning Electron Microscopy

**Figure 3:**
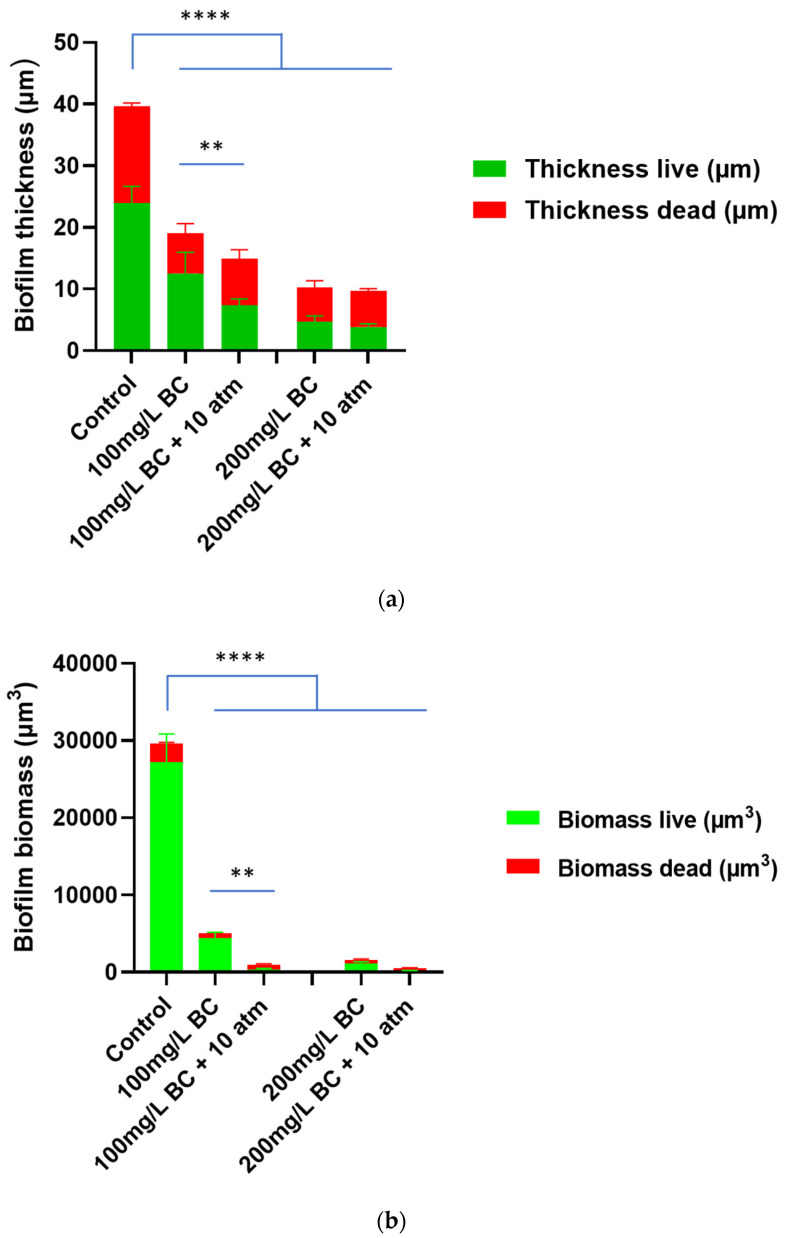
The effect of 10 min benzalkonium chloride (BC) treatment without or with 10 atm positive pressure on *S. aureus* biofilm thickness (**a**) and biomass (**b**). ** *p* < 0.01, **** *p* < 0.0001.

**Figure 4:**
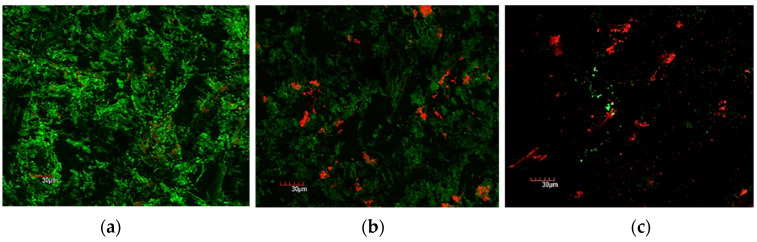
CLSM images of stained *S. aureus* biofilm with LIVE/DEAD^®^ BacLight™ Bacterial Viability Kit. Live bacteria are stained green and dead bacteria are stained red. (**a**) Control without any treatment; (**b**) treated with 10% IUC (200 mg/L) of benzalkonium chloride only for 10 min; (**c**) treated with 10% IUC (200 mg/L) of benzalkonium chloride + 10 atm of PP for 10 min.

**Figure 5:**
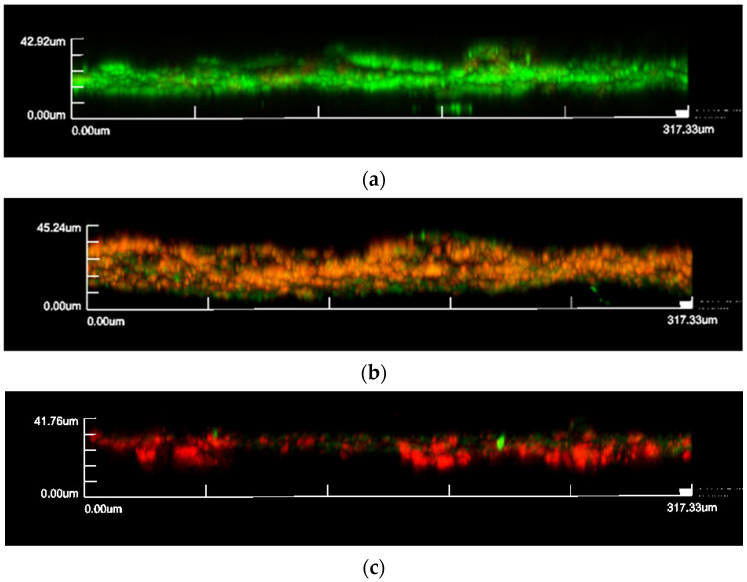
3D CLSM images of stained *S. aureus* biofilm with LIVE/DEAD^®^ BacLight™ Bacterial Viability Kit. Live bacteria are stained green and dead bacteria are stained red. (**a**) Control without any treatment; (**b**) treated with 10% IUC (200 mg/L) of benzalkonium chloride only for 10 min; (**c**) treated with 10% IUC (200 mg/L) of benzalkonium chloride + 10 atm of PP for 10 min.

Both biofilm thickness and biomass were reduced after being treated with 5% IUC (100 mg/L) or 10% IUC (200 mg/L) benzalkonium chloride for 10 min. Applying 10 atm of PP during the 10 min benzalkonium chloride treatment further reduced the biofilm thickness and biomass. The addition of 10 atm of PP also enhanced the killing of bacteria cells (Figure 3, Figure 4 and Figure 5).

**Figure 6:**
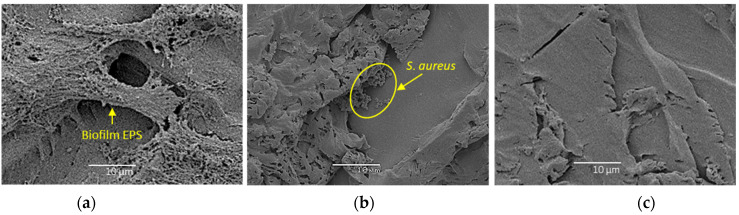
Scanning electron microscopy images (magnification 1000×) showing relative biofilm on (**a**) untreated control; (**b**) 10 min treatment with 10% IUC (200 mg/L) of benzalkonium chloride only; (**c**) 10 min treatment with 10% IUC (200 mg/L) of benzalkonium chloride + 10 atm of PP.

SEM visually confirmed the biofilm viability results obtained by CFU and CLSM. Fewer cocci in the biofilm can be seen after the 10 min treatment with 10% IUC (200 mg/L) of benzalkonium chloride and 10 atm of PP (Figure 6c) when compared to 10% IUC (200 mg/L) of benzalkonium chloride only ((Figure 6b) or untreated *S. aureus* biofilm (Figure 6a).

## 4. Discussion

### 4.1. Key Findings of the Study

The current study demonstrates that synergism between benzalkonium chloride and the application of a mild increase in pressure is possible. Benzalkonium chloride used at 10% IUC (200 mg/L) (sublethal dose) killed 4.27 logs more bacteria in the *S. aureus* biofilm when 10 atm of PP was applied over 10 min. The enhanced killing of biofilm cells was significantly seen with all treatments of 5% and 10% IUC by using 3 to 10 atm of PP for only 10 min. Further diluting benzalkonium chloride QAC (2% and 1% IUC) showed less of an impact of PP on bacterial killing, even upon extending the PP treatment time to 60 min. No significant clinically relevant effect on biofilm eradication was seen at lower concentrations of disinfectants.

### 4.2. How Does the Positive Pressure Improve the Disinfectant Killing of Biofilm Cells?

Bacteria are known to be some of the most resilient primitive living organisms on earth. Inside biofilms, they are protected by thick exopolysaccharides (EPS) and can be up to 1500 times more tolerant/resistant to antibiotics [19]. Biofilms also show increased tolerance to many disinfectants [10,20,21]. Previous scientific studies found that a 5-log reduction in CFU resulted when planktonic *S. aureus* was treated with 10−20 mg/L of benzalkonium chloride for 5 min while a much higher dose of 2000 mg/L of benzalkonium chloride produced a similar reduction when treating biofilm *S. aureus* cells. In the current study, biofilm cell killing was enhanced by 5 logs using a sub-lethal dose (200 mg/L) of benzalkonium chloride for 10 min when 10 atm of PP was added.

Previously, benzalkonium chloride efflux-resistant pumps have been identified in some *S. aureus* strains encoded by two gene family sets. The qacA and qacB genes set encodes for high resistance while another gene set with qacC and qacD genes induces low-level resistance against benzalkonium chloride and ethidium bromide [22]. A whole-genome analysis of the *S. aureus* strain ATCC 25923 used in this study demonstrated the absence of these qac resistance genes [23], hence intrinsic genetic resistance to benzalkonium chloride in *S. aureus* was ruled out. Therefore, the increased tolerance of biofilm cells is thought to be due to the biofilm lifestyle of which the thick biofilm EPS is a major component. EPS forms a barrier, thus hindering disinfectant diffusion into the biofilm. Benzalkonium chloride causes cell lysis by the physical disruption and solubilization of bacterial cell membranes and cell wall structures. However, to do this, benzalkonium chloride and other disinfectants need to contact the bacterial cell to kill it, so if disinfectants are unable to diffuse deep enough into the biofilm, cells deep in the biofilm would remain alive.

Previously, Ngo et al. demonstrated the physical disruption of an in-vitro grown *P. aeruginosa* biofilm due to compression using negative pressure wound therapy [16]. Consequently, the biofilm thickness was reduced, resulting in decreased diffusion distance and improved killing of *P. aeruginosa* biofilm cells with silver ions [16]. Synergist inactivation of methicillin-resistant *S. aureus*, *S. epidermidis,* and *P. aeruginosa* biofilm cells was also demonstrated between compression pressure and silver ions [24]. A similar phenomenon is observed in our study after applying 10 atm of PP where more than a 55% reduction was seen in live *S. aureus* biofilm thickness, while the percentage of dead cells increased from 34% to 72% when 10 atm of PP was added to the 10 min treatment with 10% IUC of benzalkonium chloride. We strongly propose that the PP forces benzalkonium chloride through the physically disrupted biofilm and thus contacts more bacterial cells for the improved kill, which is demonstrated by a two-fold higher dead cell mass in the *S. aureus* biofilm after combined treatment. With the further dilution of the benzalkonium chloride disinfectant, biofilm killing was reduced, which can be explained by the reduced availability of chemicals for disinfection. Therefore, a plateau effect was seen with 10 min of PP treatments after diluting benzalkonium chloride (2% and 1%) owing to decreased availability of the chemical and its sluggish diffusion.

### 4.3. Practical Implications and Future Directions

High positive pressure alone is currently used to decontaminate food while disinfectants are used to sterilize contaminated surfaces and equipment [18]. Some medical equipment and certainly human tissue could not withstand the higher pressure. This in vitro study was looking at positive pressure as proof of concept that slightly increased positive pressure in conjunction with antiseptics or disinfectants increased perfusion into the biofilm and thus increased killing. Our study establishes synergism between mild PP and disinfectants to microbial decontamination focusing on biofilm eradication. To combat biofilm tolerance, heat-sensitive equipment, such as endoscopes, tourniquets, and other medical equipment with hard-to-reach surfaces, can be decontaminated in a pressure chamber using the combined mild PP and disinfectants at in-use concentrations.

Due to funding restrictions, this study has only tested benzalkonium chloride against *S. aureus* biofilms. Future studies investigating the effect of mild positive pressure with other anti-biofilm agents against both hydrated biofilms and dry surface biofilms, and biofilms of other bacterial species, are warranted.

## Data Availability

Data available on request.

## References

[1] Hu H., Johani K., Gosbell I.B., Jacombs A.S., Almatroudi A., Whiteley G.S., Deva A.K., Jensen S., Vickery K. (2015). Intensive care unit environmental surfaces are contaminated by multidrug-resistant bacteria in biofilms: Combined results of conventional culture, pyrosequencing, scanning electron microscopy, and confocal laser microscopy. J. Hosp. Infect..

[2] Costa D., Johani K., Melo D., Lopes L., Lima L.L., Tipple A., Hu H., Vickery K. (2019). Biofilm contamination of high-touched surfaces in intensive care units: Epidemiology and potential impacts. Lett. Appl. Microbiol..

[3] Lopes L.K.O., Costa D.M., Tipple A.F.V., Watanabe E., Castillo R.B., Hu H., Deva A., Vickery K. (2019). Surgical instruments complex design as a barrier for cleaning effectiveness, favouring biofilm formation. J. Hosp. Infect..

[4] Hensley D.M., Krauland K.J., McGlasson D.L. (2010). *Acinetobacter baumannii* and MRSA contamination on reusable phlebotomy tourniquets. Clin. Lab. Sci..

[5] Maki D.G. (2014). Mayo Clinic: Proceedings: Stethoscopes and health care-associated infection. Mayo Clin. Proc..

[6] Stewart P.S., Franklin M.J. (2008). Physiological heterogeneity in biofilms. Nat. Rev. Microbiol..

[7] Leung C.Y., Chan Y.C., Samaranayake L.P., Seneviratne C.J. (2012). Biocide resistance of Candida and Escherichia coli biofilms is associated with higher antioxidative capacities. J. Hosp. Infect..

[8] Vickery K., Pajkos A., Cossart Y. (2004). Removal of biofilm from endoscopes: Evaluation of detergent efficiency. Am. J. Infect. Control..

[9] Da Costa Luciano C., Olson N., Tipple A.F., Alfa M. (2016). Evaluation of the ability of different detergents and disinfectants to remove and kill organisms in traditional biofilm. Am. J. Infect. Control..

[10] Otter J.A., Vickery K., Walker J.T., deLancey Pulcini E., Stoodley P., Goldenberg S.D., Salkeld J.A., Chewins J., Yezli S., Edgeworth J.D. (2015). Surface-attached cells, biofilms and biocide susceptibility: Implications for hospital cleaning and disinfection. J. Hosp. Infect..

[11] Vickery K., Ngo Q.D., Zou J., Cossart Y.E. (2009). The effect of multiple cycles of contamination, detergent washing, and disinfection on the development of biofilm in endoscope tubing. Am. J. Infect. Control..

[12] Da Costa Luciano C., Olson N., DeGagne P., Franca R., Tipple A.F.V., Alfa M. (2016). A new buildup biofilm model that mimics accumulation of material in flexible endoscope channels. J. Microbiol. Methods.

[13] Parvin F., Hu H., Whiteley G.S., Glasbey T., Vickery K. (2019). Difficulty in removing biofilm from dry surfaces. J. Hosp. Infect..

[14] Almatroudi A., Gosbell I.B., Hu H., Jensen S.O., Espedido B.A., Tahir S., Glasbey T.O., Legge P., Whiteley G., Deva A. (2016). *Staphylococcus aureus* dry-surface biofilms are not killed by sodium hypochlorite: Implications for infection control. J. Hosp. Infect..

[15] Almatroudi A., Hu H., Deva A., Gosbell I.B., Jacombs A., Jensen S.O., Whiteley G., Glasbey T., Vickery K. (2015). A new dry-surface biofilm model: An essential tool for efficacy testing of hospital surface decontamination procedures. J. Microbiol. Methods.

[16] Ngo Q.D., Vickery K., Deva A.K. (2012). The effect of topical negative pressure on wound biofilms using an in vitro wound model. Wound Repair Regen..

[17] Tahir S., Malone M., Hu H., Deva A., Vickery K. (2018). The Effect of Negative Pressure Wound Therapy with and without Instillation on Mature Biofilms In Vitro. Materials.

[18] Bello E.F., Martínez G.G., Ceberio B.F., Rodrigo D., López A.M. (2014). High Pressure Treatment in Foods. Foods.

[19] Zmantar T., Kouidhi B., Miladi H., Bakhrouf A. (2011). Detection of macrolide and disinfectant resistance genes in clinical *Staphylococcus aureus* and coagulase-negative staphylococci. BMC Res. Notes.

[20] Treangen T.J., Maybank R.A., Enke S., Friss M.B., Diviak L.F., Karaolis D.K., Koren S., Ondov B., Phillippy A.M., Bergman N.H. (2014). Complete Genome Sequence of the Quality Control Strain *Staphylococcus aureus* subsp. aureus ATCC 25923. Genome Announc..

[21] Uruén C., Chopo-Escuin G., Tommassen J., Mainar-Jaime R.C., Arenas J. (2020). Biofilms as Promoters of Bacterial Antibiotic Resistance and Tolerance. Antibiotics.

[22] Bridier A., Briandet R., Thomas V., Dubois-Brissonnet F. (2011). Resistance of bacterial biofilms to disinfectants: A review. Biofouling.

[23] Corcoran M., Morris D., De Lappe N., O’Connor J., Lalor P., Dockery P., Cormican M. (2014). Commonly used disinfectants fail to eradicate *Salmonella enterica* biofilms from food contact surface materials. Appl. Environ. Microbiol..

[24] Valente P.M., Deva A., Ngo Q., Vickery K. (2016). The increased killing of biofilms in vitro by combining topical silver dressings with topical negative pressure in chronic wounds. Int. Wound J..

